# Comparison of two inverse planning algorithms for cervical cancer brachytherapy

**DOI:** 10.1002/acm2.13195

**Published:** 2021-02-24

**Authors:** Qi Fu, Yingjie Xu, Jing Zuo, Jusheng An, Manni Huang, Xi Yang, Jiayun Chen, Hui Yan, Jianrong Dai

**Affiliations:** ^1^ Department of Radiation Oncology National Cancer Center/National Clinical Research Center for Cancer/Cancer Hospital Chinese Academy of Medial Sciences and Peking Union Medical College Beijing China

**Keywords:** cervical cancer, brachytherapy, inverse planning, IPSA, HIPO

## Abstract

**Purpose:**

To compare two inverse planning algorithms, the hybrid inverse planning optimization (HIPO) algorithm and the inverse planning simulated annealing (IPSA) algorithm, for cervical cancer brachytherapy and provide suggestions for their usage.

**Material and methods:**

This study consisted of 24 cervical cancer patients treated with CT image‐based high‐dose‐rate brachytherapy using various combinations of tandem/ovoid applicator and interstitial needles. For fixed catheter configurations, plans were retrospectively optimized with two methods: IPSA and HIPO. The dosimetric parameters with respect to target coverage, localization of high dose volume (LHDV), conformal index (COIN), and sparing of organs at risk (OARs) were evaluated. A plan assessment method which combines a graphical analysis and a scoring index was used to compare the quality of two plans for each case. The characteristics of dwell time distributions of the two plans were also analyzed in detail.

**Results:**

Both IPSA and HIPO can produce clinically acceptable treatment plans. The rectum D_2cc_ was slightly lower for HIPO as compared to IPSA (*P* = 0.002). All other dosimetric parameters for targets and OARs were not significantly different between the two algorithms. The generated radar plots and scores intuitively presented the plan properties and enabled to reflect the clinical priorities for the treatment plans. Significant different characteristics were observed between the dwell time distributions generated by IPSA and HIPO.

**Conclusions:**

Both algorithms could generate high‐quality treatment plans, but their performances were slightly different in terms of each specific patient. The clinical decision on the optimal plan for each patient can be made quickly and consistently with the help of the plan assessment method. Besides, the characteristics of dwell time distribution were suggested to be taken into account during plan selection. Compared to IPSA, the dwell time distributions generated by HIPO may be closer to clinical preference.

## 1 INTRODUCTION

Brachytherapy (BT) is an essential part of radiotherapy for locally advanced cervical cancer (LACC). Nowadays, three‐dimensional(3D) image‐guided radiotherapy has been widely used in BT.^1^ With the integration of 3D images (CT, MRI), the dose distribution can be adjusted to fit the individual anatomical situation. The conventional treatment planning approach is to manually activate source positions and manually adjust dwell times for better target coverage and sparing of organs at risk (OARs). This is an iterative forward planning method which requires an experienced planner to spend a lot of time changing the dwell weights constantly until an optimal solution is met. Over the last two decades, inverse planning has been more applied to BT.^2^ It is based on mathematical optimization algorithms, which was commonly used in external beam radiotherapy (EBRT). The principle of an inverse planning optimization algorithm is to search for the minimum value of an aggregate objective function based on a set of predefined dose objectives. Compared with forward planning, inverse planning has advantages including less planning time, better reproducibility, higher target coverage, and lower dose to OARs.^3^, ^4^, ^5^, ^6^

At present, two of the most common inverse planning methods available in commercial practice are: inverse planning simulated annealing (IPSA) and hybrid inverse planning optimization (HIPO). IPSA is a general algorithm which optimizes the source dwell times using a fast simulated annealing stochastic algorithm.^7^, ^8^, ^9^, ^10^, ^11^ The optimization process takes no more than 1 minute. HIPO is an optimization algorithm proposed more recently, which combines the simulated annealing stochastic algorithm and the limited memory Broyden‐Fletcher‐Goldfarb‐Shanno (LBFGS) deterministic algorithm for 3D dose distribution optimization.^5^, ^13^, ^14^ Manual source position activation and partial catheter optimization are permitted in HIPO. Both algorithms were initially developed for prostate cancer BT and have been fully demonstrated by several groups.^15^, ^16^, ^17^, ^18^ However, for cervical cancer BT, the clinical application of inverse planning is still not widespread, due to the small number of catheters and the limitation of catheter placement. Recently, the IPSA optimizer was improved by adding a special parameter to restrict the dwell time variance between adjacent dwell positions in a catheter.^19^ Its effects are still under investigation. As an alternative to IPSA, HIPO also has not been fully studied.^5^, ^20^ So far, only Ref. 21 has compared HIPO with un‐improved IPSA. Thus, it is necessary to investigate the algorithms with constraint optimization and more clinical cases in order to make better use of them for cervical cancer BT especially with small number of catheters.

For a given patient, different optimization algorithms may result in comparable plans. A radiation oncologist needs to determine quickly which of them is the optimal plan for treatment. Although there are many dosimetric parameters and quality indexes that can be used for plan comparison, it becomes complicated when the large and diverse amount of data are analyzed. The clinical decision made by radiation oncologists is time‐consuming and easily based on subjective and qualitative assessment of the planned dose distributions considering only the most important features of the plan. Although several studies have proposed methods and tools for quantitative comparison of multiple plans,^22^, ^23^, ^24^, ^25^, ^26^ they are mostly focused on EBRT and have never been adopted in BT. There is a lack of effective methods to compare BT plans quantitatively, comprehensively and consistently.

This study compared the dosimetric outcomes and characteristics of dwell time distributions for the plans generated using IPSA and HIPO for cervical cancer BT. A special plan assessment method was applied for quantitative comparison of quality among different treatment plans.

## 2 MATERIAL AND METHODS

Twenty‐four patients treated between January 2017 and December 2019 were selected from our institution’s clinical database for this retrospective study. According to FIGO stage classification,^27^ the local tumor stage of the patients was as follows: IB2 = 2, IIB = 6, IIIB = 3, IIIC1r = 8, IIIC2r = 5. All the patients underwent 45 to 50 Gy whole pelvic EBRT followed by five fractions of intracavitary/interstitial brachytherapy (IC/ISBT) with prescribed dose (PD) of 6 Gy. Nucletron standard tandem/ovoid (T/O) applicators and interstitial needles were used to deliver the IC/ISBT treatment. According to the different tumor shapes, the patients were treated with different combinations of applicators and needles as follows: seven patients with one tandem two ovoids, seven with one tandem three needles, seven with one tandem two ovoids two needles, two with one tandem two ovoids three needles, one with one tandem two ovoids four needles. After the insertion of applications, all patients underwent CT scans using the Brilliance CT Big Bore (Philips, Amsterdam, Netherlands) with 3‐mm slice thickness. These scans were transferred to the Oncentra Brachy v4.6 (Elekta Brachytherapy, Veneedal, The Netherlands), where high‐risk clinical target volume (HR CTV), intermediate‐risk clinical target volume (IR CTV), bladder, rectum, sigmoid, and bowel were contoured in accordance with GEC ESTRO recommendation.^28^, ^29^ The HR CTVs covered a wide range, between 22.6 and 140.8 cc (mean 68.0 cc). IR CTV was a 3‐mm volumetric expansion of HR CTV while subtracting all OARs. We treated it as a target but also as a help structure to control high dose regions outside HR CTV during dose optimization. The dose volume constraints in this study followed NCCN clinical practice guidelines v. 3.2019 (see Table 1).^30^ The median of the constraint ranges and the EBRT dose of 45 Gy/25f were adopted for determining the dose volume constraints per fraction of IC/ISBT. Direct applicator reconstruction was carried out on the CT images using multi‐planar reconstruction (MPR). All treatment plans were planned using the Oncentra Brachytherapy planning system v4.6, with a ^192^Ir source for a Flexitron afterloader unit. The activation step was set to 2 mm.

**TABLE 1:** The dose‐volume constraints used for this study (Gy).

	Total^1^	One fraction of IC/ISBT
HR CTV D_90_	≥80‐87	≥6
Bladder D_2cc_	≤80‐90	≤5.1
Rectum D_2cc_	≤65‐75	≤4.3
Sigmoid D_2cc_	≤70‐75	≤4.1
Bowel D_2cc_	‐	≤4.3

^1^ The equivalent accumulated dose of EBRT and IC/ISBT at 2 Gy (EQD2).

### 2.1 IPSA planning

IPSA provides a combination of source activation, dose normalization, dose optimization, and dose prescription. Thus, the optimization can be performed just after contouring and applicator reconstruction. Table 2 shows the initial optimization settings used in this study. HR CTV was identified as the Reference Target. Note that its minimum surface/volume doses (700 cGy) were set to be higher than the PD just for optimization, aiming to increase the coverage of the targets while keeping the dose to OARs unchanged as far as possible. When the plan was optimized, the dose to 90% of HR CTV (D_90_) would be normalized to 100% of the PD (600 cGy). The optimization parameters were adjusted and the calculation was repeated if the clinical objective was not achieved.

**TABLE 2:** Dose volume objectives used for the IPSA plans.

Contour	Min (cGy)	Weight	Max (cGy)	Weight
HR CTV (surface)	700	200	1500	10
HR CTV (volume)	700	200	2500	1
IR CTV (surface)	500	10	800	50
IR CTV (volume)	500	10	1500	20
Bladder (surface)			430	50
Rectum (surface)			400	30
Sigmoid (surface)			400	30
Bowel (surface)			400	30

In Oncentra Brachy v4.3 and above, the IPSA optimization engine introduced a special parameter, dwell time deviation constraint (DTDC), which allows restriction of the difference in dwell times between adjacent dwell positions within each catheter. The DTDC value can be set from 0.0 to 1.0, where 0 is an unrestricted optimization and 1 is a homogeneous plan. Using DTDC can avoid the presence of isolated positions with extremely large dwell times. But studies have shown that a high value of DTDC may work against target coverage and OARs sparing.^19^, ^31^ In this study, the DTDC was set to 0.1.

### 2.2 HIPO planning

HIPO was only used for the optimization of the dose distribution in this study, hence the source dwell positions were set the same as those for IPSA. The optimization parameters are listed in Table 3. IR CTV and HR CTV were identified as the PTV and GTV, respectively. Similar to DTDC, the dwell time gradient restriction (DTGR) is a modulation restriction parameter for HIPO to restrict large fluctuations between dwell times in neighboring dwell positions. It is also a relative value between 0.0 and 1.0, reflecting the “weight” of its importance in the optimization solution space.^31^ The higher the value, the smaller the fluctuation.^32^ However, to minimize adverse impact on target coverage and OARs sparing, the DTGR was set to 0.1 as well. Moreover, HIPO enables manual control of the sampling point settings for regions of interest (ROIs). For a high optimization precision, we increased the number of sampling points proportionally to the volumes of targets and OARs.

**TABLE 3:** Dose volume objectives used for the HIPO plans.

Contour	Min (% PD)	Weight	Max (% PD)	Weight	Priority
HR CTV	140	100	500	0.1	5
IR CTV	100	10	200	50	6
Bladder			80	70	1
Rectum			70	50	2
Sigmoid			70	50	3
Bowel			70	50	4
Normal tissue			100	1	‐

### 2.3 Plan evaluation

The dose volume parameters recommended by GEC ESTRO GYN were analyzed for all plans, including D_90_ (dose to 90% of HR CTV and IR CTV), V _CTV,200_ (the volume of HR CTV receiving 200% of the PD), D_2cc_ (minimal dose received by the most irradiated 2 cc volume of bladder, rectum, sigmoid and bowel). The conformity index (COIN) was used to evaluate how well the PD covers the target volume and excludes nontarget volumes, which was calculated as follows: ^33^

(1) $$\text{COIN}=V_{\text{CTV},\text{ref}}^{\text{2}}/\left(V_{\text{CTV}}\times V_{\text{ref}}\right),$$

where V_CTV,ref_ is the volume of CTV that receives dose equal to or greater than PD; V_ref_ is the volume receiving the PD. As the high dose region is a cause of concern, we defined a factor, localization of high dose volume (LHDV), to characterize how accurately the high dose regions are localized inside of HR CTV. The LHDV is the ratio of V_CTV,200_ to V_200_ (the total volume receiving 200% of the PD). In addition, the dwell time distribution and the proportion of tandem loading time in total loading time (T_tan/tot_) were also analyzed.

For each patient, the plan assessment method described in Ref. 22 was adopted to quantitatively compare which of the two plans has a better performance. The method consists of two parts. The first is a graphical analysis providing a set of radar plots to show each quality score intuitively. The second is a total plan score weighting all quality scores to evaluate plan quality entirely. The quality score of each dosimetric parameter mentioned above can be calculated according to the following expression:

(2) $$S_{j}=\left\{\begin{array}{c} \frac{C_{j}}{P_{j}},\;\;\;\text{for targets}\\ \frac{P_{j}}{C_{j}},\;\;\;\text{for OARs} \end{array}\right),$$

where *C*_j_ is the constraint value of objective *j* given in Table 4, and *P*_j_ is the corresponding plan value. For targets, a high *P*_j_ represents a high coverage, homogeneity or conformal index, resulting in a low *S*_j_. Similarly, for OARs, a low *S*_j_ means a low dose to the OAR. Each quality score is represented by a point along the angle bisector of the corresponding objective in the radar plot. The distance between the point and the radar plot center corresponds to the score value. By connecting all the points, a polygon representing the plan quality is generated. The smaller the polygon area, the higher the plan quality.

**TABLE 4:** The scoring parameters used in the plan assessment method.

Objective	Constraint	Weight
Bladder D_2cc_ (Gy)	≤5.1	15%
Rectum D_2cc_ (Gy)	≤4.3	20%
Sigmoid D_2cc_ (Gy)	≤4.1	20%
Bowel D_2cc_ (Gy)	≤4.3	15%
IR CTV D_90_ (Gy)	≥4	10%
HR CTV COIN	1	10%
LHDV	1	5%
Total loading time (s)	≤400*	5%

* The value was chosen only to obtain a quality score and show it in radar plot, with no actual meaning.

The total plan score was defined as follows:

(3) $$S_{\text{total}}=\sum_{j}w_{j}\cdot S_{j},$$

where *w*_j_ is the weight of objective *j*, which reflects its importance in clinical treatment. As shown in Table 4, the weights used for this study were defined by a group of two professional treatment planners and three radiation oncologists based on clinical practice. The set of weights represented our clinical preferences. The two‐sided paired t‐test was used to make statistical comparisons of different quality indices between the IPSA and HIPO plans.

## 3 RESULTS

**FIG. 1:**
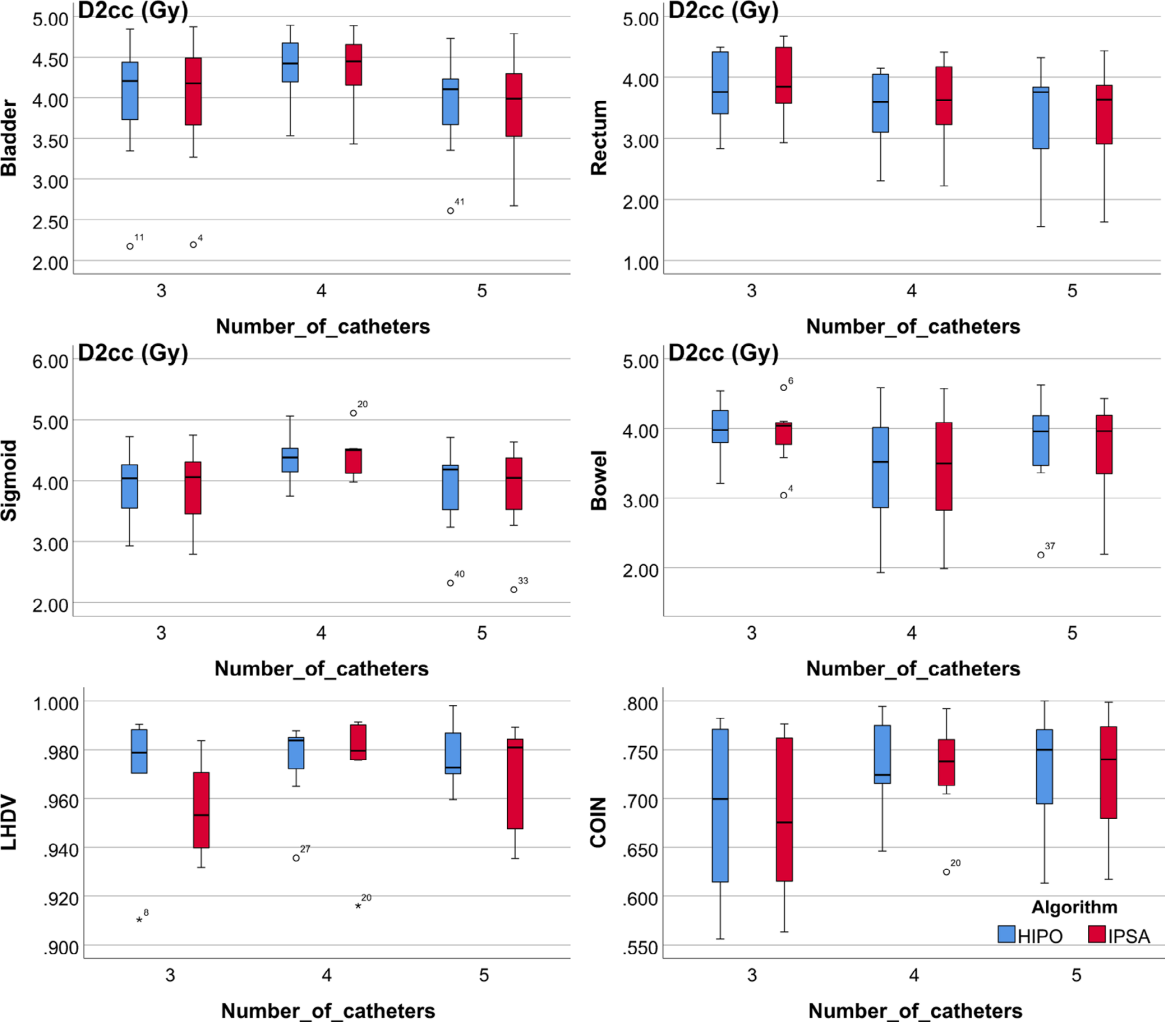
Box plots of the main dosimetric parameters (DHI and COIN for HR CTV, D_2cc_ for bladder, rectum, sigmoid, and bowel) for the IPSA and HIPO plans with different number of catheters.

**FIG. 2:**
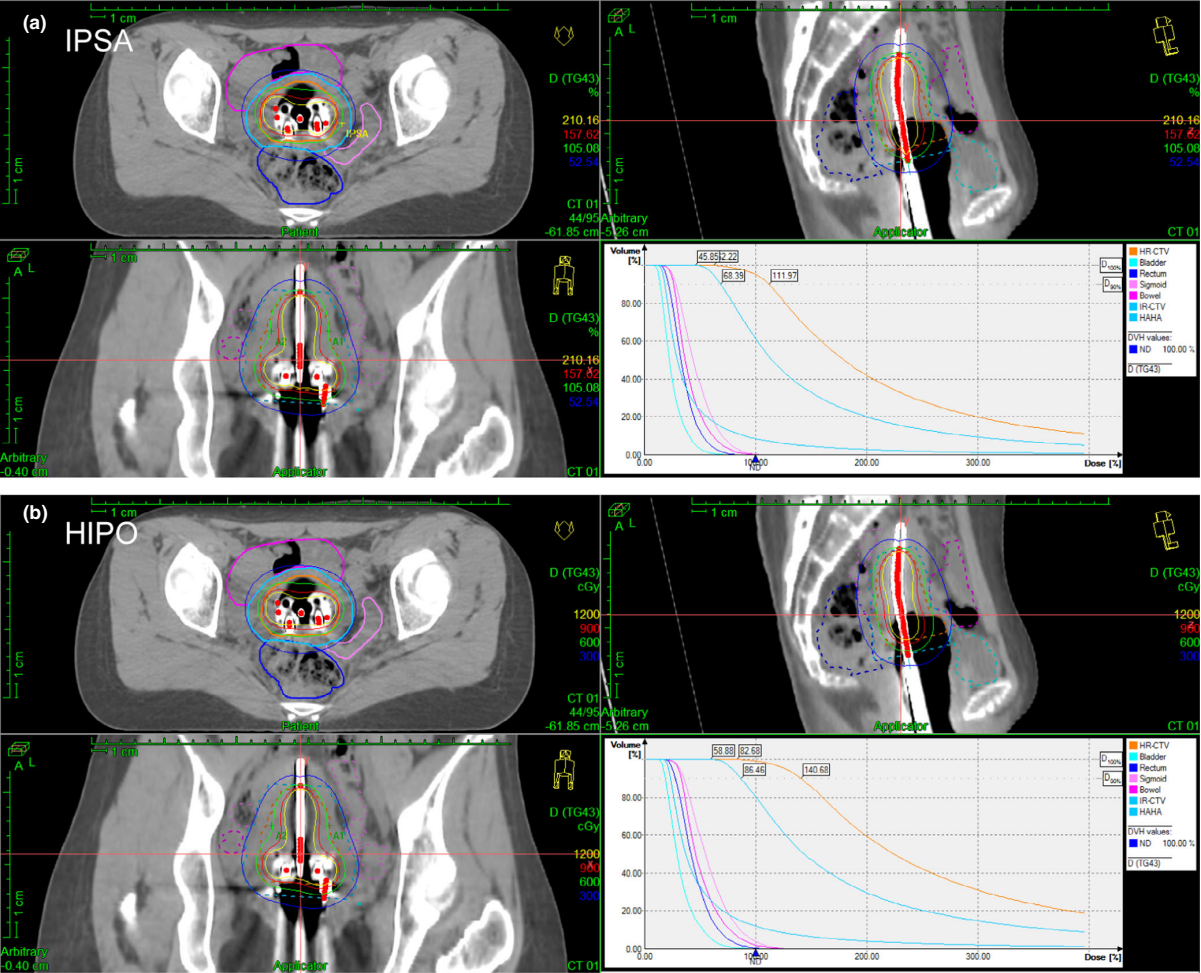
Typical dose distributions of the IPSA (a) and HIPO (b) plans in the axial, coronal, sagittal, and DVH views.

Both IPSA and HIPO were able to produce dosimetrically acceptable treatment plans. Table 5 shows the mean values and standard deviations of dosimetric parameters together with the *t*‐values and the statistical significances (*p*‐values) of all compared parameters for the two plans. No significant difference was observed with dosimetric parameters for HR CTV and IR CTV as well as the bladder, sigmoid and bowel between the two plans. The rectum D_2cc_ for HIPO was only 0.08 Gy lower than that for IPSA, although with a *p*‐value of 0.002. The average value of LHDV was slightly higher for HIPO when compared with IPSA (*p* = 0.09), which may be because some dwell times for the IPSA plans often occurred close to both ends of the activated dwell positions while that for the HIPO plans was more concentrated in the middle of the catheter (see Fig. 5). Figure 1 presents similar results in the form of box plots. It also can be seen that there is no significant relationship between the main dosimetric parameters with the number of catheters, either for the IPSA plans or for the HIPO plans. An example of typical dose distribution after the HIPO and IPSA optimizations are shown in Figure 2. Both plans were created for the same patient treated by combining the T/O applicator with four interstitial needles.

**TABLE 5:** Comparison of dosimetric parameters between the IPSA and HIPO plans.

	IPSA	HIPO	*t*	*P*
HR CTV D_90_ (Gy)	6.00 ± 0.00	6.01 ± 0.00	1.622	0.118
IR CTV D_90_ (Gy)	4.00 ± 0.54	4.03 ± 0.51	1.355	0.188
HR CTV COIN	0.712 ± 0.067	0.717 ± 0.069	1.444	0.162
LHDV	0.955 ± 0.071	0.966 ± 0.046	1.723	0.098
Bladder D_2cc_ (Gy)	3.96 ± 0.74	3.97 ± 0.72	0.623	0.540
Rectum D_2cc_ (Gy)	3.56 ± 0.77	3.48 ± 0.74	3.55	0.002
Sigmoid D_2cc_ (Gy)	3.99 ± 0.70	3.98 ± 0.66	0.220	0.828
Bowel D_2cc_ (Gy)	3.55 ± 0.85	3.59 ± 0.85	1.386	0.179

**FIG. 3:**
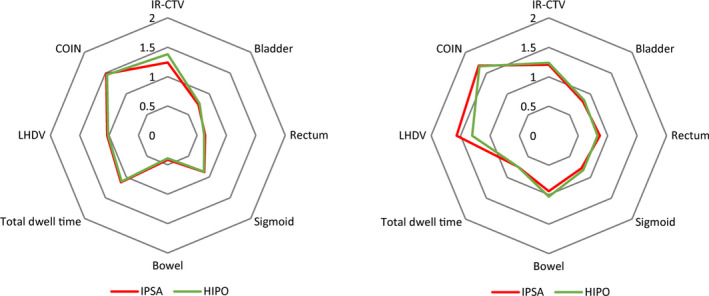
Radar plots of two examples showing the quality scores for the IPSA and HIPO plans.

**FIG. 4:**
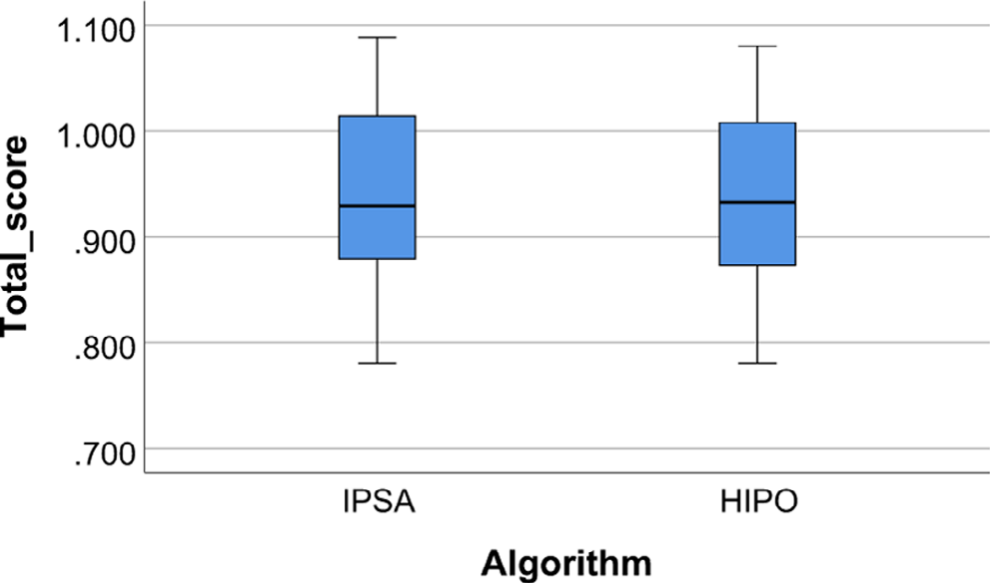
Total plan scores for the IPSA and HIPO plans in the form of box plots.

Figure 3 presents two radar plots corresponding to two cases of the study. The innermost octagon of the plots represents the constraints of all the objective, and out of the octagon implies exceeding the constraints, by which planners or radiation oncologists would be able to easily analyze the plan properties. When the polygon is closer to the plot center, the corresponding plan is superior. Thus, for the left plot, the IPSA plan is better than the HIPO plan and for the right plot, the opposite is true. In addition to the radar plot, the total weighted score can be used for plan comparison more directly. That is, a lower score corresponds to a better plan. Figure 4 compared the total scores between the IPSA and HIPO plans. The IPSA plan scores were lower than the HIPO plan scores for eight among the 24 patients. Mean total scores for IPSA and HIPO were 0.948 ± 0.082 and 0.939 ± 0.079, respectively, with a *P*‐value of 0.020. This may indicate that although difference exists in each single case, the two algorithms present comparable performances as a whole.

**FIG. 5:**
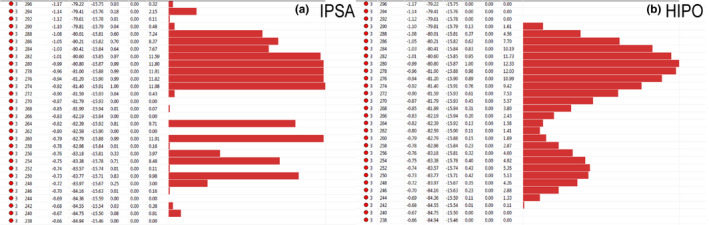
Example of dwell times distribution as calculated by IPSA with DTDC = 0.1 (a) and HIPO with DTGR = 0.1 (b) for the same patient and within the same catheter.

The total loading time and the proportion of tandem loading time for the IPSA and HIPO plans were compared in Table 6. Still, their values show no significant differences between the two plans (*p* = 0.937, 0.812). However, obvious different characteristics were observed from the dwell time distributions generated by the two algorithms. As shown in Figure 5a, the dwell times calculated by the IPSA algorithm have an inhomogeneous distribution. There were large fluctuations between dwell times in neighboring dwell positions, resulting in some dwell positions with very long times while others with short times or empty. Figure 5b shows that the dwell times obtained using the HIPO algorithm formed a wave distribution, and changes in the neighboring dwell times were continuous and smooth.

**TABLE 6:** Comparison of dwell times between the IPSA and HIPO plans.

	Total loading time (s)	T_tan/tot_
IPSA	380 ± 173	0.588 ± 0.164
HIPO	381 ± 174	0.591 ± 0.151
*t*	0.080	0.241
*P*	0.937	0.812

## 4 DISCUSSION

With the advantage in target coverage and normal tissue sparing, inverse optimization techniques have been gradually replacing manual optimization for high‐dose‐rate (HDR) BT, particularly for prostate BT. For cervix BT, inverse planning algorithms, especially the IPSA algorithm, have been implemented by several institutions and positive results have been published.^3^, ^4^, ^5^, ^6^, ^7^, ^8^, ^9^, ^10^, ^12^ Recent clinical results further verified the use of IPSA in the clinics.^34^, ^35^ Kim et al. and Tinkle et al. successively concluded that IPSA‐planned HDR BT is well tolerated with minimal toxicities and achieves excellent local control. As inverse optimization advanced, the HIPO algorithm was proposed in 2005,^13^ and then a parameter restricting dwell time variance has been added to the IPSA optimization module of Oncentra Brachy v4.3 in 2013.^19^ However, so far, there is little study on the IPSA and HIPO algorithms for cervical cancer BT. Only Trnková et al. concluded that HIPO was superior in the elimination of high dose regions in normal tissue.^21^

This study that compared the two inverse planning algorithms was based on the T/O applicator and interstitial needles, due to the limited conditions for anesthesia and relatively narrow vagina of most female patients in China. It is worth pointing out that the T/O applicator has relatively fewer numbers of catheters and dwell positions compared with interstitial template, multi‐channel cylinder or tandem/ring applicator that are commonly used in Europe and America. The reported experiences and optimization methods are limited and insufficient. Therefore, we made several improvements in the planning process to achieve a high level of plan quality. For both IPSA and HIPO plans, we set the lower dose constraints of HR CTV to be higher than the PD before optimization and lowered the D_90_ of HR CTV to the PD after optimization. This may be helpful for a better target coverage without compromising the OARs sparing. As an expansion of HR CTV, the IR CTV was utilized as a help structure in this study. Adding maximum dose objectives to it can significantly increase the dose conformity to HR CTV and effectively restrict high dose regions outside HR CTV. The maximum dose objectives to HR CTV were relatively loose, considering a certain volume of high dose region was acceptable inside the treated volume for cervical cancer. As reported in Ref. 20 and 36, HIPO allows optimizing the intracavitary applicator and the interstitial needles separately to increase the proportion of dwell time of tandem in total time and reduce hot spots around needles. However, as the number of dwell positions for our cases was limited, the separate optimization with iterative approach had more limited degree of freedom for dwell time optimization. For some cases, it was even hard to obtain an optimal plan. Therefore, the needles and T/O were optimized at the same time with equal weighting to increase the degree of freedom for the HIPO optimization. In addition, the settings of sampling points for dose optimization are fully automated in IPSA but manually adjustable in HIPO. Most studies on HIPO did not mention the sampling points or set them as defaults. However, we found that an inadequacy of sampling points may cause a decline of the plan quality. Thus, the number of sampling points was set to be changed according to the different volumes of OARs and targets.

The most striking difference between IPSA and HIPO is in dwell time distribution, due to the use of DTDC and DTGR parameters (see Fig. 5). Both parameters are modulation restrictions of dwell times in their respective optimization modules but quite different in principle.^19^, ^31^, ^32^ The DTDC parameter in IPSA defines a dwell time upper limit to control the dwell time variations between adjacent dwell positions in each catheter. Whereas the DTGR parameter in HIPO is a dwell time gradient objective to restrict large dwell time fluctuations in neighboring dwell positions. As mentioned earlier, a large DTDC or DTGR may overly restrict the dwell time distribution and limit the optimization, thereby affecting the plan quality. But without dwell time constraints, in some cases, the dwell times would present an extremely inhomogeneous distribution in which some individual positions have very long dwell times while others have short or zero dwell times. This could produce the spatial dose distribution that conform to the specific shape of the target and minimize the doses to OARs, but also lead to extremely high dose regions. A few investigators have noticed that this had the potential risk of inducing toxicity if there is a displacement of the catheters.^37^, ^38^, ^39^ The displacement of a large dwell time apparently has a greater effect on the treatment plan than if dwell time differences were not so significant. Furthermore, the current clinical practice is mainly based on the traditional loading systems and the impact of deviation from the traditional loading pattern is still unclear. Therefore, large differences between dwell times (just as those generated by IPSA) are usually not clinically acceptable. Although the difference of dwell time distributions between the two algorithms barely reflected on plan quality, HIPO was able to produce a smoother dwell time distribution, which may result in the dose distributions in more rounded shape and were closer to the clinically ideal distribution. Whereas the dwell times generated by IPSA, especially the very short or long dwell times, probably need to be manually modified before they could be implemented for treatment.

In this study, a plan assessment method used for EBRT was applied to BT. This method aims to deal with the problems related to ranking and selection of treatment plans generated using different algorithms, treatment techniques or treatment planning systems. For instance, it is proved above that the IPSA and HIPO algorithms would be able to produce comparable plans, but how to assess and determine which is better for an individual patient is a complicated and time‐consuming issue for radiation oncologists. Although the modified COIN used in some studies can quantify target coverage as well as normal tissue sparing,^40^, ^41^ it does not consider clinical demands or preferences and the information provided are still not comprehensive for plan evaluation. The plan assessment method adopted in this article provided not only the radar plot to present the plan quality intuitively but also the total plan score to integrate all the quality scores weighted according to clinical preferences. With the help of the plan assessment method, our medical team made decisions much easier and faster and avoided personal choice. The clinical decisions on the best plan were also made consistently when comparing different plans. However, it is worth noting that the plan score is just an adjuvant tool provided for plan comparison and is no substitute for clinical decision. Both the objectives and their weights listed in Table 4 were based on our own clinical experience, for reference only. If using this plan score, it is necessary to follow local clinical practice and complete clinical test to suit different situations or demands.

## 5 CONCLUSION

Two different inverse optimization algorithms (IPSA and HIPO) for cervical cancer BT were compared in this study. The plan qualities resulted by the two algorithms were comparable as a whole but slightly different for each individual patient. Using the plan assessment method is recommended to make a fast and consistent decision in selecting the optimal plan taking into account all clinical priorities and criteria. Besides, the characteristics of dwell time distribution also could be considered as one of the influences on clinical decision. Compared to IPSA, HIPO could generate smoother dwell time distribution, which may result in more clinically desirable dose distribution.

## AUTHOR CONTRIBUTIONS

Conception and design of the study – Qi Fu, Yingjie Xu, Jing Zuo. Acquisition and collection of data – Qi Fu, Jusheng An, Manni Huang, Xi Yang. Assessment of treatment planning ‐ Jusheng An, Manni Huang. Analysis and interpretation of data ‐ Qi Fu, Yingjie Xu, Jiayun Chen. Writing and revising the paper ‐ Qi Fu, Yingjie Xu, Hui Yan, Jiayun Chen. Final approval of the manuscript ‐ Yingjie Xu, Jianrong Dai.

## CONFLICT OF INTEREST

No conflict of interests.
